# Knowledge about Alzheimer’s disease in medical, nursing, and psychology students in Ecuador: A problem that needs an urgent solution

**DOI:** 10.1371/journal.pone.0350624

**Published:** 2026-06-01

**Authors:** José Alejandro Valdevila Figueira, María Alejandra Espinoza de los Monteros Andrade, Xavier Rodrigo Yambay-Bautista, Andrés Ramírez, Indira Dayana Carvajal Parra, Rocío Valdevila Santiesteban, María José Pico, Jose A. Rodas

**Affiliations:** 1 Universidad Ecotec, Samborondón, Ecuador; 2 Red de Investigación en Psicología y Psiquiatría (RIPYP), Ecuador; 3 Instituto de Neurociencias, Junta de Beneficencia de Guayaquil, Ecuador; 4 Carrera de enfermería, Universidad Católica de Cuenca, Azogues, Ecuador; 5 Escuela de Psicología, Universidad Espíritu Santo, Samborondón, Ecuador; 6 School of Psychology, University College Dublin, Dublin, Ireland; Universidade dos Açores Departamento de Biologia: Universidade dos Acores Departamento de Biologia, PORTUGAL

## Abstract

**Background:**

Alzheimer’s disease represents one of the greatest healthcare challenges of the 21st century due to the aging population and its impact on the quality of life of patients and their families. Preparing future healthcare professionals to address this condition is crucial.

**Aims:**

This article analyzes the level of knowledge about Alzheimer’s disease held by university students in medicine, nursing, and psychology, highlighting the differences and similarities between disciplines and proposing strategies to improve training in this field.

**Methods:**

A cross-sectional study was conducted with a convenience sample of 1,023 Ecuadorian students: nursing (n = 727, 71.1%), medicine (n = 170, 16.6%), and psychology (n = 126, 12.3%). Participants completed the Alzheimer’s Disease Knowledge Scale (ADKS) and a demographic survey. The percentage of correct answers on the ADKS was used to assess knowledge levels.

**Results:**

The overall percentage of correct answers was 54.68%, indicating a limited level of knowledge. Medical students obtained the highest mean score (17.44 [SD: 2.864]), followed by psychology (16.28 [SD: 2.348]) and nursing (16.18 [SD: 2.649]). A weak but significant correlation was found between knowledge level and prior contact with people with dementia (P < 0.001).

**Conclusions:**

Students across all disciplines demonstrated a broad knowledge gap regarding Alzheimer’s disease, although medical students obtained slightly higher scores than psychology and nursing students. The findings highlight the need for improved educational training and curriculum development to enhance dementia knowledge, especially in psychology and nursing programs.

## Introduction

### Global and national context of Alzheimer’s disease

Alzheimer’s disease (AD) is a progressive neurodegenerative disease that primarily affects older people, characterized by cognitive decline, memory loss, and behavioural changes [1]. It is the most common form of dementia and one of the leading causes of disability in old age, with nearly 10 million people are diagnosed each year as new cases [2], disproportionately affecting women, both directly and indirectly, who also have higher rates of disability-adjusted life years and mortality from this disease [3].

The estimated global cost of dementia is equivalent to approximately 1.1% of global gross domestic product (GDP), with an estimated increase of approximately US$500 billion in dementia spending every 5 years [4]. In 2019, nearly 50% of the economic cost of dementia care was related to care provided by informal caregivers, such as family members and close friends [5]. The costs and prevalence of dementia appear to be associated with patterns of multimorbidity, as individuals living with dementia often experience multiple co-occurring chronic conditions that increase the complexity and intensity of care required [6].

In Latin America, the burden of dementia is increasing at an unprecedented rate due to rapid demographic transitions and an ageing population. Despite this growing epidemiological challenge, regional healthcare systems confront systemic barriers, including fragmented care pathways, limited diagnostic resources, and critical gaps in the specialised training of healthcare professionals. Consequently, evaluating and enhancing dementia education within Latin American universities is essential to ensure that future practitioners are adequately prepared to manage this escalating public health crisis.

AD accounts for between 60% and 70% of dementia cases. In Ecuador, AD affects 5% of people over 65, with an exponential increase in the following age groups, reaching 30% in those over 80 and 50% in those over 90 [6] and around 59,000 people live with AD, in addition to nearly half a million Ecuadorians being directly linked to the disease [7].

Given its increasing prevalence, comprehensive care involving professionals from multiple health disciplines is required [8–10]. Therefore, it is essential to assess and strengthen the knowledge of university students training in fields such as medicine, nursing, and psychology. In this regard, several studies reveal a lack of knowledge about AD among nursing students [11], psychology and medicine [12].

According to the estimate of the Ecuadorian Institute of Statistics and Census (INEC) [9] around 15% of Ecuadorians over the age of 60 suffer from dementia. The average longevity of Ecuadorians (78 years) ranks third in South America and rose from 74th in 2021–48th worldwide in 2022, meaning that people with dementia can live a long life. The older adult population currently represents 9% of the national population and is expected to increase rapidly in the coming decades [9].

### Knowledge and training of health sciences students

In this context, adequate knowledge about AD allows future professionals to recognize early signs and symptoms, understand the clinical course of the disease, promote patient-centered care strategies, and address the emotional and social impact on caregivers and family members [13]. However, some studies have shown that there is variability in the level of knowledge depending on the field of study, which may compromise multidisciplinary care [14].

Medical students often have a deeper understanding of the pathophysiology of AD, thanks to their neuroscience training. However, they may show deficiencies in the psychosocial approach and in communication skills with patients and family members, essential aspects in the comprehensive management of the disease [13].

Nursing education emphasizes ongoing patient care and attention. Students in this discipline tend to demonstrate greater practical knowledge of the daily management of patients with AD, including techniques for effective communication and behavioural symptom management [15]. However, their understanding of the underlying biological mechanisms may be more limited compared to medical students [16].

Psychology students tend to have a solid understanding of the behavioural and emotional changes associated with AD [13]. In addition, they are trained to intervene in supporting patients and their caregivers, despite possible regional limitations found in their training across countries (e.g., [17]). Their understanding of neurobiological aspects may be less detailed, but their training in cognitive assessment and emotional support is essential for a comprehensive approach [18].

Comparative studies reveal that medical and psychology students show greater theoretical knowledge [19] and nursing staff excel in practical aspects of care [20]. Furthermore, there is a shared need to improve training in patient-centered care, interdisciplinary work, and the ethical approach to AD.

### Rationale and aim of the study

The literature reviewed recommends developing integrated curricula that incorporate AD content across all disciplines, adapting them to the professional approach, promoting interdisciplinary educational interventions through workshops, seminars, and joint simulations across disciplines, including supervised clinical practice that facilitates early contact with AD patients in real-life clinical settings, and emphasizing a biopsychosocial approach by strengthening the understanding of AD beyond cognitive impairment, considering social and emotional aspects. This study’s aim was to measure the level of knowledge towards Alzheimer’s disease in a sample of university students from Guayaquil, Ecuador.

## Methods

### Study design

A cross-sectional, observational, quantitative study was conducted. The sample was selected using non-probability convenience sampling.

### Participants

A total of 3,121 students from the psychology, nursing, and medicine programs at the University of Cuenca, Ecuador, were invited to participate via institutional emails. A total of 1,421 (45.5%) responded to the invitation. A survey was sent to the participants to collect demographic data. A total of 398 records were excluded due to incomplete data. The final sample included 1,023 students, distributed by field of study: nursing (n = 727; 71.1%), medicine (n = 170; 16.6%), and psychology (n = 126; 12.3%).

Institutional email was used as the primary recruitment strategy to ensure the survey reached the target population while maintaining adherence to university data privacy protocols.

### Instruments

The Alzheimer’s Disease Knowledge Scale (ADKS) assesses knowledge in seven domains: risk factors, symptoms, evaluation/diagnosis, disease course, impact on life, treatment/management, and care. The Ecuadorian Spanish-validated version of the ADKS by Ramírez-Coronel, Jorge et al. was used [21].

In addition, a demographic questionnaire was administered to collect information on sex, marital status, place of residence, ethnic self-identification, university degree, previous contact with people with dementia, family history of cognitive impairment, and living with people diagnosed with dementia.

### Procedure

Data collection took place between 11/03/2024 and 01/04/2024–56 virtual sessions with approximately 25 participants in each session. During the sessions, the demographic questionnaire and the ADKS scale were completed. Participant confidentiality and anonymity were guaranteed; responses were securely stored in an encrypted and anonymised system.

This study was conducted in compliance with the ethical standards outlined in the Helsinki Declaration of 1975, as revised in 2013. All procedures involving human subjects were approved by the Research Bioethics Committee of the Health Area at the University of Cuenca (Approval Code: 2022–011EO-IE). Before data collection, all participants provided electronic informed consent and completed the demographic questionnaire and the ADKS scale. Ethical guidelines, including confidentiality, voluntary participation, and data protection, were strictly followed. Data collection was administered via Google Forms, with the survey settings explicitly configured to ensure anonymity by disabling the collection of email addresses and precluding the capture of personally identifiable information. Following data collection, the anonymised dataset was exported and securely stored on a password-protected, encrypted institutional server. Access to these electronic records was strictly restricted to the principal investigator, ensuring comprehensive adherence to the reproducible data management protocols mandated by the research bioethics committee.

### Analysis

Univariate analysis was performed to obtain frequencies and percentages for categorical variables, as well as measures of central tendency and dispersion for numerical variables. The Kolmogorov-Smirnov test confirmed that the data did not follow a normal distribution, so Spearman's correlation coefficient (ρ) was used to assess the relationship between ADKS scores and sample characteristics (P < 0.05).

Multinomial logistic regression was performed to identify predictors of Alzheimer's knowledge level, using the group with the highest knowledge level as the reference category. The model included regression coefficients (B), standard errors, Wald statistics, and odds ratios (OR) with 95% confidence intervals and p values (< 0.05). Model fit was assessed using Nagelkerke's R² coefficient and the chi-square test (p < 0.05). Statistical analyses were performed using Jamovi v2.3.28 and SPSS® v29.0 (trial version).

## Results

### Demographic descriptive data

The sample consisted of 1,023 university students, distributed among the medical (n = 170), nursing (n = 727), and psychology (n = 126) programs. The median age was 22 years (IQR = 4), with slight variations between programs: 21 years in nursing, 22 in medicine, and 23 in psychology.

Regarding gender, women predominated (74.0%) in the total sample, especially in nursing (79.9%) and psychology (60.0%). Medicine showed a more balanced distribution, with 40.0% male participants.

The majority of students identified themselves as single (90.7%), with this proportion being highest in medicine (94.7%). Regarding place of residence, 67.8% lived in urban areas, with medicine standing out with 82.4% urban residents, while nursing had a higher proportion of students from rural areas (36.6%).

Regarding ethnic self-perception, 94.9% identified themselves as mestizo, followed by minority groups such as Indigenous (3.7%), Afro-Ecuadorians (0.6%), and whites (0.8%). Mestizo identification was most prevalent in psychology (98.4%) and medicine (97.6%).

In terms of academic progress, students were mainly distributed in the sixth semester (19.5%), with an absolute predominance in medicine (80.6%). In nursing and psychology, greater diversity was observed in the semester distribution, including a significant representation of students in the internship period (26.4% in nursing, 0% in the other programs).

Regarding experience with people with dementia, 9.6% of students reported having had previous contact, with no significant differences between programs. A further 13.1% reported a family history of cognitive impairment, with this proportion being highest in medicine (17.6%). Finally, 3.7% reported currently living with a family member with dementia, with the highest proportion in psychology (7.1%). Table 1 presents the particiapans’characteristics.

**Table 1:** Characteristics of the participants according to study area.

Variable	Total N = 1,023	Medicine (n = 170)	Nursing (n = 727)	Psychology (n = 126)
**Median age** (IQR)	22 (4,00)	21 (2,00)	22 (3,00)	23 (4,00)
**Gender**				
Male	266 (26,0%)	68 (40,0%)	147 (20,2%)	51 (40%)
Female	757 (74,0%)	102 (60,0%)	580 (79,88%)	75 (60%)
**Marital status**				
Single	928 (90,7%)	161 (94,7%)	653 (89,8%)	114 (90,5%)
Married/Cohabiting	82 (8,0%)	9 (5,3%)	62 (8,5%)	11 (8,7%)
Separated/Divorced	13 (1,3%)	0 (0,0%)	12 (1,7%)	1 (0,8%)
**Residence**				
Urban	694 (67,8%)	140 (82,4%)	461 (63,4%)	93 (73,8%)
Rural	329 (32,2%)	30 (17,6%)	266 (36,6%)	33 (26,2%)
**Self-perceived ethnicity**				
Mestizo	971 (94,9%)	166 (97,6%)	681 (93,7%)	124 (98,4%)
Indigenous	38 (3,7%)	3 (1,8%)	35 (4,8%)	0 (0,0%)
Afro-Ecuadorian	6 (0,6%)	0 (0,0%)	5 (0,7%)	1 (0,8%)
White	8 (0,8%)	1 (0,6%)	6 (0,8%)	1 (0,8%)
**Semester**				
First	62 (6,1%)	6 (3,5%)	49 (6,7%)	7 (5.6%)
Second	105 (10,3%)	3 (1,8%)	91 (12,5%)	11 (8,7%)
Third	131 (12,8%)	0 (0,0%)	109 (15,0%)	22 (17,5%)
Fourth	96 (9,4%)	1 (0,6%)	69 (9,5%)	26 (20,6%)
Fifth	112 (10,9%)	6 (3,5%)	85 (11,7%)	21 (16,7%)
Sixth	199 (19,5%)	137 (80,6%)	45 (6,2%)	17 (13,5%)
Seventh	72 (7,0%)	1 (0,6%)	65 (8,9%)	6 (4,8%)
Eighth	11 (1,1%)	4 (2,4%)	0 (0,0%)	7 (5.6%)
Ninth	6 (0,6%)	3 (1,8%)	0 (0,0%)	3 (2,4%)
Tenth	37 (3,6%)	9 (5,3%)	22 (3,0%)	6 (4,8%)
Internship	192 (18,8%)	0 (0,0%)	192 (26,4%)	0 (0,0%)
**Contact with people with dementia**				
No	925 (90,4%)	156 (91,8%)	657 (90,4%)	112 (88,9%)
Yes	98 (9,6%)	14 (8,2%)	70 (9,6%)	14 (11,1%)
**Family history of cognitive impairment**				
No	889 (86,9%)	140 (82,4%)	640 (88,0%)	109 (86,5%)
Yes	134 (13,1%)	30 (17,6%)	87 (12,0%)	17 (13,5%)
**Lives with a relative with dementia**				
No	985 (96,3%)	165 (97,1%)	703 (96,7%)	117 (92,9%)
Yes	38 (3,7%)	5 (2,9%)	24 (3,3%)	9 (7,1%)

This table displays the demographic and academic characteristics of the study participants, categorized by field of study (medicine, nursing, and psychology). Variables include age, gender, marital status, place of residence, ethnicity, academic level, prior contact with individuals with dementia, family history of cognitive impairment, and cohabitation with a relative diagnosed with dementia.

*Note.* IQR = interquartile range*.*

**Fig 1:**
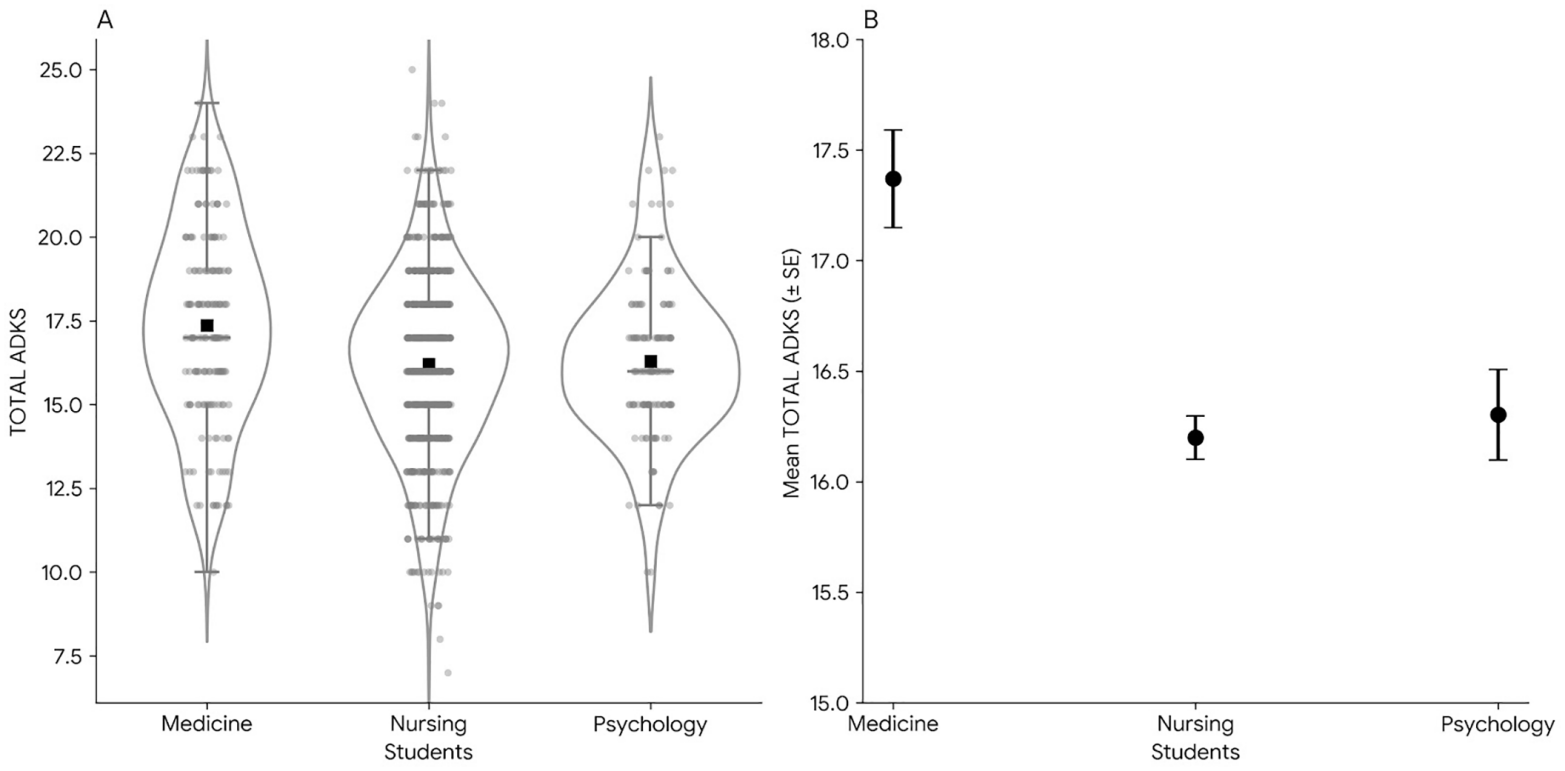
Distribution and mean differences of the alzheimer’s disease knowledge scale total score across academic programmes. Note. Panel A illustrates the data distribution of total Alzheimer’s Disease Knowledge Scale (ADKS) scores using violin plots, box plots, and individual data points to depict variability and central tendency. Panel B presents the mean total ADKS scores for each academic programme, with error bars indicating the standard error of the mean.

The overall ADKS score allowed for the evaluation and comparison of AD knowledge across different fields of study. The overall percentage of correct answers was 54.68%, indicating a moderate level of knowledge about AD. Medical students achieved the highest average score (17.44 [SD, 2.864]), followed by psychology students (16.28 [SD, 2.348]) and nursing students (16.18 [SD, 2.649]). Fig 1 illustrates the distribution of scores by speciality, highlighting those medical students exhibited the greatest variability, whereas nursing students demonstrated a more centred and consistent distribution.

A one-way ANOVA with Welch’s correction revealed statistically significant differences in total ADKS scores across academic programmes, F(2, 267) = 11.85, p < .001, η^2^ = .026. Descriptively, medical students obtained the highest mean score (n = 173, M = 17.40, SD = 2.90, SE = 0.22), followed by psychology (n = 129, M = 16.30, SD = 2.33, SE = 0.21) and nursing students (n = 738, M = 16.20, SD = 2.64, SE = 0.10). Games-Howell post hoc comparisons confirmed that medical students scored significantly higher than nursing (mean difference = 1.17, t = 4.86, df = 243, p < .001, d = .43) and psychology students (mean difference = 1.07, t = 3.54, df = 298, p = .001, d = .42). No statistically significant differences were observed between nursing and psychology students (mean difference = −0.10, t = −0.45, df = 190, p = .893, d = .04). Overall, these findings indicate that medical students possess a greater overall knowledge of Alzheimer’s disease compared to the other two disciplines, which demonstrated comparable performance.

### Dementia knowledge by dimension

Seven dimensions of dementia knowledge were assessed: risk factors, symptoms, diagnosis, course of disease, impact on life, treatment, and care (see Table 2). The overall percentage of correct answers varied across dimensions, with the treatment (65.96%) and course of disease (65.32%) categories showing the highest performance, while the “impact on life” dimension had the lowest percentage (39.82%).

**Table 2:** ADKS scores, according to subdimensions and specialty (N = 1,023).

			Group of students		
			Medicine	Nursing	Psychology
Dimension	Mean/SD	% correct	Mean/SD	Mean/SD	Mean/SD
Risk factors	2,93 ± 1,194	48,76	2,84 ± 1,253	2,93 ± 1,197	3,00 ± 1,088
Symptoms	2,30 ± 0,972	57,43	2,59 ± 1,058	2,20 ± 0,935	2,45 ± 0,968
Diagnosis	2,52 ± 0,847	62,88	2,49 ± 0,823	2,54 ± 0,861	2,38 ± 0,788
Course of the disease	2,61 ± 0,921	65,32	2,91 ± 0,905	2,58 ± 0,917	2,40 ± 0,878
Impact on life	1,19 ± 1,123	39,82	1,25 ± 1,114	1,19 ± 1,119	1,15 ± 1,160
Treatment	2,64 ± 0,893	65,96	2,98 ± 0,835	2,58 ± 0,908	2,49 ± 0,767
Care	2,22 ± 0,913	44,4	2,39 ± 0,886	2,15 ± 0,921	2,40 ± 0,849

This table presents the mean scores and standard deviations for each subdimension of ADKS, categorized by field of study (medicine, nursing, and psychology). The percentage of correct answers is also reported for each subdimension, indicating the areas where students demonstrated greater or lesser knowledge.

*Note.* SD = standard deviation.

Regarding the overall averages by dimension, the following means (± standard deviation) were observed: risk factors (2.93 ± 1.19), symptoms (2.30 ± 0.97), diagnosis (2.52 ± 0.85), course of illness (2.61 ± 0.92), impact on life (1.19 ± 1.12), treatment (2.64 ± 0.89), and care (2.22 ± 0.91).

When broken down by program, notable differences were identified. Medical students obtained higher scores in the treatment (2.98 ± 0.84) and course of illness (2.91 ± 0.91) dimensions, while psychology students excelled in risk factors (3.00 ± 1.09) and symptoms (2.45 ± 0.97). In contrast, nursing students showed more homogeneous scores across all dimensions, with particularly low performance in the “impact on life” dimension (1.19 ± 1.12).

In the care dimension, psychology students obtained the highest mean (2.40 ± 0.85), followed by medicine (2.39 ± 0.89) and nursing (2.15 ± 0.92). The differences between groups suggest possible curricular variations in the approach to dementia, depending on the discipline.

### Correlations between demographic characteristics and knowledge about dementia

Spearman’s correlation coefficient was applied to explore the relationship between various demographic variables and the level of knowledge about dementia. Results are presented in Table 3.

**Table 3:** Correlation analysis, ADKS vs demographic variables (N = 1,023).

Variable	Spearman’s Rho	p-value	Interpretation
Age	0,063	0,043	Weak positive and significant correlation
Gender	0,010	0,739	Not significant
Marital status	0,043	0,174	Not significant
Place of Residence	−0,030	0,332	Not significant
Self-perceived Ethnicity	−0,041	0,187	Not significant
Specialty	−0,133	< 0,001	Weak negative and significant correlation
Semester	0,013	0,678	Not significant
Contact with People with Dementia	0,076	0,015	Weak negative and significant correlation
Family History of Cognitive Impairment	0,113	< 0,001	Weak negative and significant correlation
Lives with a Relative with Dementia	0,040	0,201	Not significant

This table presents the results of the Spearman’s rank correlation analysis between ADKS scores and various demographic variables, including age, gender, marital status, place of residence, self-perceived ethnicity, specialty, semester, prior contact with people with dementia, family history of cognitive impairment, and cohabitation with a relative with dementia. The correlation coefficient (Spearman’s Rho), p-value, and interpretation of statistical significance are reported.

A weak but statistically significant positive correlation was observed between participants’ age and knowledge about dementia (ρ = 0.063; p = 0.043), suggesting that knowledge tends to increase slightly with age. In contrast, the variable “academic major” showed a weak but significant negative correlation (ρ = −0.133; p < 0.001), indicating that knowledge levels vary by major, with lower levels of knowledge in some disciplines.

Furthermore, previous contact with people with dementia was positively associated with knowledge (ρ = 0.076; p = 0.015), as was having a family history of cognitive impairment (ρ = 0.113; p < 0.001), suggesting that personal experience may contribute to a greater understanding of the topic.

Other variables, such as gender (ρ = 0.010; p = 0.739), marital status (ρ = 0.043; p = 0.174), place of residence (ρ = −0.030; p = 0.332), ethnic self-perception (ρ = −0.041; p = 0.187), semester completed (ρ = 0.013; p = 0.678), and living with a family member with dementia (ρ = 0.040; p = 0.201), did not show statistically significant correlations with knowledge level.

These findings suggest that personal experiences related to dementia, such as contact or family history, are associated with greater knowledge, while differences between specialties could reflect curricular variations in training on the subject.

### Factors associated with dementia knowledge level

A multinomial logistic regression analysis was performed using high levels of dementia knowledge as the reference category (results are presented in Table 4). The model identified several statistically significant predictors associated with a lower likelihood of belonging to the high-knowledge group.

**Table 4:** Multinomial logistic regression analysis (N = 1,023).

Predictor (category)	B	SE	Wald	OR (95% CI)	*p*
**Low knowledge level vs high knowledge level**					
Specialty (Medicine)	−3,271	1,641	3,975	0,038 (0,002−0,946)	**0,046**
Semester (Fifth)	−3,188	1,362	5,447	0,041 (0,003−0,596)	**0,019**
**Medium knowledge level vs high knowledge level**					
Specialty (Medicine)	− 1,662	0,700	5,629	0,190 (0,048−0,749)	**0,018**
Contact with people with dementia (NO)	1,668	0,452	13,630	5,302 (2,187−12,854)	**<0,001**

This table presents the results of a multinomial logistic regression analysis examining predictors of ADKS scores. The model compares low and medium knowledge levels against high knowledge levels, identifying significant predictors such as specialty (medicine), semester (fifth), and prior contact with people with dementia. Reported values include the regression coefficient (B), standard error (SE), Wald statistic, odds ratio (OR) with 95% confidence intervals (CI), and statistical significance (p).

*Note.* B = regression coefficient; OR = Odds ratio; CI = confidence interval; p = statistical significance. Model Indicators: R² Nagelkerke = 0.193, Chi-square = 82.082 (p = 0.001).

Comparing the low-knowledge group versus the high-knowledge group, it was observed that medical students were less likely to have a low level of knowledge compared to students from other specialties (B = −3.271; OR = 0.038; 95% CI: 0.002–0.946; p = 0.046). Similarly, being in the fifth semester was associated with a significantly lower probability of being in the low knowledge group (B = −3.188; OR = 0.041; 95% CI: 0.003–0.596; p = 0.019), suggesting that certain levels of academic progress may be related to greater exposure to the subject.

In the comparison between the medium knowledge group and the high knowledge group, a significant association with specialty was also observed. Medical students again showed a lower likelihood of being in the medium knowledge group (B = −1.662; OR = 0.190; 95% CI: 0.048–0.749; p = 0.018). On the other hand, not having had prior contact with people with dementia was associated with a significantly higher likelihood of having a medium level of knowledge (B = 1.668; OR = 5.302; 95% CI: 2.187–12.854; p < 0.001).

Given the modest model fit (Nagelkerke R² = .19), these regression results should be considered exploratory. Interpretations regarding the influence of academic training and personal experience on dementia knowledge must remain conservative.

## Discussion

This study evaluated the level of knowledge about AD among health sciences students at an Ecuadorian university. The results showed a low overall level of knowledge, with an average correct answer rate of 54.68% on the ADKS scale. This finding is consistent with international studies that have indicated significant limitations in knowledge about AD among medical, nursing, and psychology students [22].

These findings provide insight into local educational gaps, however, the reliance on a single university setting in Ecuador, means this sample is not representative of the broader Latin American region, restricting the external validity and international relevance of the conclusions.

### Knowledge around the world

Other reviewed studies show wide variability in the level of knowledge about dementia among university students, especially in health-related programs, highlighting differences associated with demographic, academic, and contextual factors. For example, in Nepal, undergraduate students obtained a mean score on the ADKS of 15.45 ± 2.95, with limited knowledge and no significant association between gender and knowledge level (p = 0.71). In contrast, science students demonstrated higher knowledge than administration students (p = 0.004), indicating that academic background significantly influences the level of understanding of dementia. This pattern is repeated in studies from China, where medical students obtained higher scores than nursing students (p < 0.05), and those with higher educational levels, experience, or family members with medical knowledge tended to demonstrate better performance on the ADK [23].

In India, limited overall knowledge was found among nursing students, with only 56% correct answers and a negative correlation between age and knowledge (r = −0.323; p < 0.001). This suggests that younger students—possibly at earlier stages of their training—may have had more recent exposure to educational content on dementia.

In Turkey, despite a female predominance (82.9%) and some personal or family experience with dementia, knowledge was considered inadequate (13.01 ± 6.66 out of 34), although attitudes were moderately positive (82.53 ± 12.44). Weak but significant associations were observed between knowledge and variables such as sex, educational level, and having received formal education on dementia, highlighting the importance of specific training beyond personal experiences [24].

In contrast, nursing students in Malta demonstrated adequate knowledge, attributable to the curricular integration of the subject and practical experience during their studies. This suggests that clinical exposure to patients with dementia is a determining factor for a deeper understanding of the disease [25].

Chinese university students, although scoring slightly higher on the ADKS (18.92 ± 3.20 and 19.49 ± 2.82 in different studies), also demonstrated insufficient knowledge and less positive attitudes compared to students from developed countries. However, a positive (albeit weak) association was detected between knowledge and attitudes (r = 0.122; p = 0.028), reinforcing the idea that better education could improve not only knowledge but also perceptions towards people with dementia [12].

These findings align with international research, highlighting that knowledge deficits regarding Alzheimer’s disease are not exclusive to low- and middle-income nations. Studies conducted in high-income settings, such as the United States, the United Kingdom, and Spain, consistently report moderate to insufficient knowledge levels among health sciences students [26–28]. Research evaluating Spanish university cohorts indicates persistent gaps in subdomains such as communication and health risk promotion among nursing students compared to psychology students [26]. Similarly, evaluations in the United Kingdom have demonstrated that younger adult populations reveal significant knowledge gaps pertaining to risk factors and conditions that exacerbate vulnerability to the disease [27]. Furthermore, assessments in the United States found that although final-year medical students exhibit better theoretical understanding than their first-year counterparts, a substantial proportion still demonstrates overall knowledge deficiencies regarding patient management [28]. Consequently, the struggle to adequately prepare healthcare students for dementia care represents a global educational challenge.

### Education and knowledge

Consistent with previous research, the findings indicate a broad knowledge gap across all assessed disciplines. Medical students obtained slightly higher scores, which could be attributed to greater exposure to neuroscience, pathophysiology, and pharmacology content [29]. Despite this, its average score (17.44/30) still indicates an insufficient level of knowledge, which reinforces the need to strengthen training programs even in this career.

In contrast, nursing and psychology students showed lower general knowledge and lower levels of knowledge in several specific areas, such as diagnosis, course of illness, and treatment. These results are concerning, given the key role these professionals play in early detection, psychosocial care, and support for patients and families. Studies conducted in Latin America, such as those by Diaz-Guecha and Márquez-Delgado in Colombia [30], and Ramírez-Coronel et al. in Ecuador for 2021 [21], similar findings have been reported, suggesting a regional trend in poor education about dementia.

However, overall performance across all groups was modest, and the observed mean differences between academic disciplines, alongside the associations with demographic variables, were relatively small. Consequently, recommendations for extensive curriculum reform should be interpreted cautiously, as the practical significance of these differences remains limited.

The long-term impact of lack of knowledge about dementia and Alzheimer's has been reflected in research findings among healthcare professionals from various fields. For example, a study of healthcare professionals at a tertiary care hospital in India showed a mean ADKS score of 19.2 ± 3.1. Participants with a positive family history and experience in personal/professional AD care did not score significantly better. Physicians were more likely to score better than nurses and other healthcare staff (p < 0.001). Dementia education had a significantly greater impact on promoting AD knowledge (p < 0.001) [31]. Research involving experienced nursing staff in Jordanian hospitals reported similarly deficient knowledge scores, indicating that knowledge deficits regarding AD represent a persistent issue across the training continuum rather than being an issue exclusive to the undergraduate student population.

Higher levels of education (semesters completed) and medical specialization (medical students) are associated with greater knowledge about AD [12,14,15,23]. Although people with medical education may have better comprehension skills to understand complex health problems, other studies suggest that the level of education does not directly affect knowledge about AD [13,30,32]. Furthermore, undergraduate learning programs can enhance knowledge and collaborative skills, as demonstrated by a study by O'Sullivan et al. [32]. This study used a learning workshop for clinical practice students and found a statistically significant increase in students’ knowledge and confidence levels when communicating with a person with dementia after the training. Exposure to practical experiences and the use of innovative educational resources can significantly improve the competence and empathy of future healthcare professionals in caring for people with dementia.

To address these educational deficits, universities should implement interprofessional education frameworks that facilitate collaborative learning among medical, nursing, and psychology students. Integrating experiential learning models, such as supervised clinical rotations in dementia care units and simulated patient encounters, is crucial. These pedagogical strategies transition students from theoretical understanding to practical application, fostering the empathy, communication skills, and interdisciplinary teamwork required for effective, patient-centred dementia care.

Demographic factors positively associated with knowledge level were identified in our study, such as previous contact with people with dementia and a family history of cognitive impairment. As in some other studies, this research did not reveal a significant relationship between marital status and levels of knowledge of AD. On the contrary, a study conducted in the general population in Northern Ireland found statistically significant differences between marital status and levels of knowledge about AD. In general, the disparities in these results could be related to demographic factors, cultural and religious attitudes towards marriage [33] and health training [34].

### Limitations

Despite its contributions, this study has several limitations that should be considered when interpreting the findings. First, the cross-sectional design restricts the ability to establish causal relationships between demographic or experiential variables and levels of knowledge about Alzheimer’s disease. Second, the use of a non-probability convenience sample limits the representativeness of the results, as the findings cannot be generalized to all medical, nursing, or psychology students in Ecuador or other contexts. Furthermore, the sample exhibited a highly unbalanced distribution across academic programmes, with nursing students comprising the majority, alongside an overrepresentation of women and individuals of mestizo ethnicity. These structural biases limit the ability to conduct meaningful comparative analyses between disciplines and restrict the generalisability of the findings. Also, the reliance on email-based recruitment introduces potential self-selection and non-response biases. Because data collection was completely anonymous, it was not possible to conduct comparative analyses between responders and non-responders.

Third, the Alzheimer’s Disease Knowledge Scale (ADKS), although validated in Ecuador and widely used internationally, relies on a dichotomous true/false format that may inflate or underestimate actual knowledge due to guessing effects. In addition, self-reported data on prior contact with individuals with dementia may be subject to recall or social desirability bias. Finally, this study focused on a single geographic and institutional context, which may not capture potential curricular or cultural variations across other regions or universities. Future research would benefit from longitudinal designs, probabilistic sampling, the inclusion of qualitative approaches to explore attitudes and perceptions, and the comparison of multiple institutions to enhance external validity.

## Conclusions

This study revealed a broad knowledge gap regarding Alzheimer’s disease among medical, nursing, and psychology students at a single university in Ecuador. Regional extrapolations must be approached with caution due to the geographically restricted and unbalanced nature of the sample. While medical students demonstrated relatively higher scores, all groups showed important gaps, particularly regarding the psychosocial impact of dementia and its implications for daily life. These results highlight the urgent need to strengthen health sciences curricula with integrated and interdisciplinary content on dementia, combining theoretical foundations with practical experiences that facilitate early exposure to patients and caregivers.

Personal experiences, such as prior contact with people living with dementia or a family history of cognitive impairment, were associated with slightly higher knowledge levels, underscoring the role of experiential learning in preparing future professionals. However, demographic variables such as sex, marital status, and academic semester showed no significant associations, suggesting that structural educational strategies may be more decisive than individual factors.

Given the rapid aging of the population and the projected rise in dementia prevalence, improving the training of future healthcare providers is both a clinical and public health priority. Incorporating dementia-specific modules, interprofessional workshops, and early clinical rotations could enhance knowledge, empathy, and preparedness to provide comprehensive care. Future research should explore the effectiveness of such interventions across multiple institutions and disciplines to better inform policy and educational reform.

Ultimately, these exploratory findings may serve as a preliminary step to inform local educational strategies. If supported by broader, representative research, such data could eventually contribute to discussions on national dementia plans in Latin America. Leveraging academic collaborations could help explore the harmonisation of dementia education standards and support future policy evaluations.
